# Finite Size Effects in Simulations of Protein Aggregation

**DOI:** 10.1371/journal.pone.0002641

**Published:** 2008-07-09

**Authors:** Amol Pawar, Giorgio Favrin

**Affiliations:** Department of Chemistry, University of Cambridge, Cambridge, United Kingdom; Emory University, United States of America

## Abstract

It is becoming increasingly clear that the soluble protofibrillar species that proceed amyloid fibril formation are associated with a range of neurodegenerative disorders such as Alzheimer's and Parkinson diseases. Computer simulations of the processes that lead to the formation of these oligomeric species are starting to make significant contributions to our understanding of the determinants of protein aggregation. We simulate different systems at constant concentration but with a different number of peptides and we study the how the finite number of proteins affects the underlying free energy of the system and therefore the relative stability of the species involved in the process. If not taken into account, this finite size effect can undermine the validity of theoretical predictions regarding the relative stability of the species involved and the rates of conversion from one to the other. We discuss the reasons that give rise to this finite size effect form both a probabilistic and energy fluctuations point of view and also how this problem can be dealt by a finite size scaling analysis.

## Introduction

A major field of study in protein science is the understanding of the causes, and implications of protein aggregation. The misfolding of proteins often results in aggregates and in the formation of highly regular structures called amyloid fibrils [1]–[3]. Amyloids are best known for their involvement in pathological conditions such as type II diabetes systemic amyloidosis and neurodegenerative disorders such as Alzheimer's, Parkinson's and Creutzfeldt-Jakob diseases [3]–[7]. Increasing evidence is showing that indeed it is the soluble pre-fibrillar or oligomeric species, rather than the insoluble amyloid fibrils themselves, which are responsible for toxicity and neuronal disfunction [8]–[10]. Even if highly complex processes are associated with oligomer toxicity, a view is gaining support according to which the ability to form toxic oligomeric species represents an intrinsic property of polypeptide chains at some stage of their oligomerization process [8], [10]. Despite this growing interest in the role of peptide and protein oligomers in disease, the molecular mechanism by which they are formed is still the object of investigation of several *in vivo*, *in vitro*, and *in silico* studies [4], [11]–[19]. However, since it is challenging to describe the early stages of aggregation of polypeptide chains experimentally, primarily because of the difficulties in detecting and characterising the small, structurally heterogeneous and transient species that are involved, a detailed description of this process at the molecular level remains in large part elusive. In particular the heterogeneous nature of these species implies that their free energy landscape is very rugged. Theoretical studies are starting to make important contributions to the understanding of these diseases by investigating the partially unfolded intermediates and the structural features of the oligomeric species [15], [16], [18], [20]–[24].

Due to limitations in computers power, simulations are usually performed in the NVT ensemble on systems ranging form a couple of peptides with detailed all atom models to several hundred with very coarse grained models [15]–[35]. We will discuss how these studies can be affected by the finite size effect and how this has repercussions on the underlying free energy of the system. This is indeed a major difference between *in silico* and *in vitro* studies, as in the latter the number of proteins is in the order of the Avogadro number. This effect has been studied in the case of proteins of different lengths [36]. In the following we will discuss in detail the finite size effect specifically in the case of simulations of protein aggregation and we will underline some strategies to deal with it.

## Results

### Free Energy Calculations in Systems with Different Sizes

We study how the actual number of proteins or peptides in our simulation, irrespectively of their concentration, affects the underlying free energy landscape of the system. In particular we investigate six systems of three, four, six, eight, nine and twelve “Gly-Phe-Phe” (GFF) peptides at constant concentration and for eight different temperatures using the parallel tempering technique and with periodic boundary conditions (see Methods). A similar peptide (Phe-Phe), was studied experimentally by the group of Gazit [37] and it was found to form nanotube-like structures. We choose however to add a Gly to the peptide to increase its propensity to form inter-peptide hydrogen bonding.

**Figure 1:**
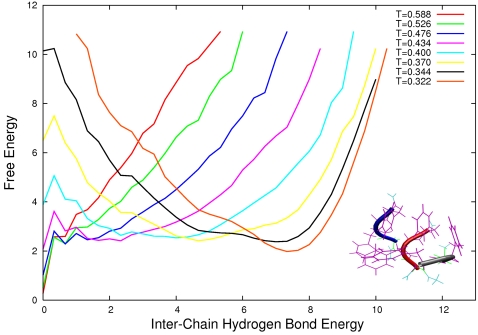
Free Energy landscape as a function of inter chain hydrogen bond energy for a system of 3 peptides for temperatures ranging from T = 0.588 to T = 0.322. In the lower right corner we show a characteristic oligomeric configuration at T = 0.322.

**Figure 2:**
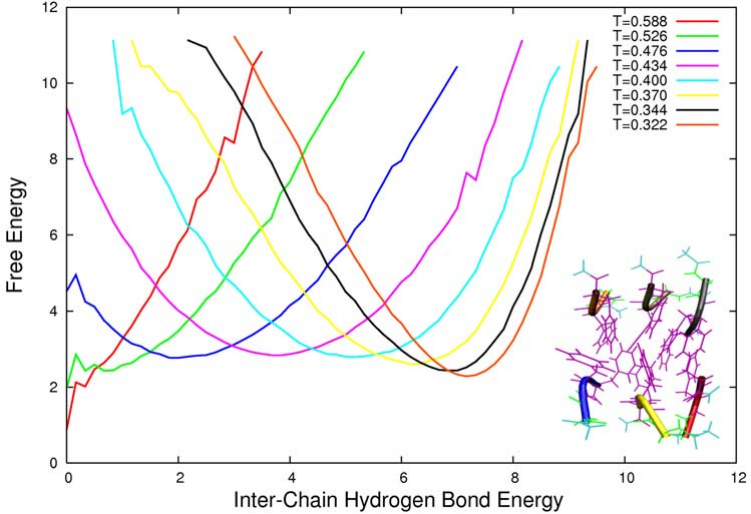
Free Energy landscape as a function of inter chain hydrogen bond energy for a system of 6 peptides for temperatures ranging from T = 0.588 to T = 0.322. In the lower right corner we show a characteristic oligomeric configuration at T = 0.322. By comparing the free energy profile for T = 0.4 in the present and in Fig: 1, we can notice the increase in stability of the aggregated phase that results by simply increasing the number of peptide in the system and the volume of the system so that the concentration remains constant.

**Figure 3:**
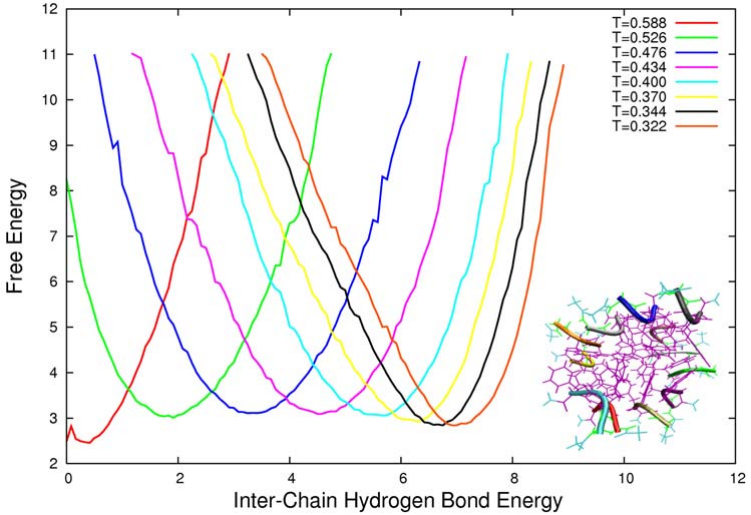
Free Energy landscape as a function of inter chain hydrogen bond energy for a system of 12 peptides for temperatures ranging from T = 0.588 to T = 0.322. In the lower right corner we show a characteristic oligomeric configuration at T = 0.322.

**Figure 4:**
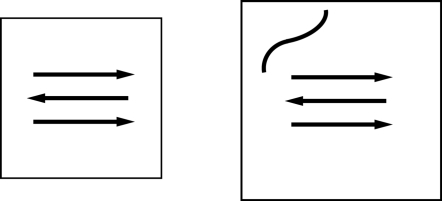
Schematic change in the probability of aggregation in two systems with different number of proteins. If the proteins in the system are all aggregated (left) the stability of an oligomer formed by three proteins is limited by the probability of one protein to go back in solution. In another system (right) both the number of proteins and the volume are increased so that the concentration is left unchanged but since there is one monomer still in solution that can potentially aggregate, the stability of the oligomer is increased.

**Figure 5:**
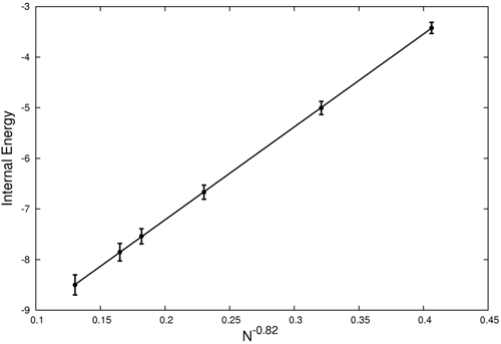
Finite size scaling analysis. We fitted the average internal energy in each system to the equation: *e*(*N*) = *e*_∞_+*AN^α^* and we plotted the results as a function of N^−0.82^, where −0.82 is the value of α obtained from the fit using all our available data.

For each system we calculate the free energy as a function of the inter-chain hydrogen bond interaction energy normalised over the number of peptides for each different temperature used in the parallel tempering. In this way we can monitor the formation and stability of the aggregated state alone. Our computationally efficient Monte Carlo sampling allow us to calculate easily the free energy landscape for systems up to twelve GFF peptides. In Fig 1, Fig 2, and Fig 3 we plot such landscapes and we can notice how in each system the aggregated state becomes more stable as the temperature decreases and how by increasing system size the aggregates become stable for increasingly higher temperatures. The influence of the number of particles on the free energy of the system for a given concentration can be explained by the following example. If we consider a system where all *N* proteins are aggregated (*e.g. N* = 3 in Fig 4, left) the probability of another protein to be added to the oligomer is zero simply because there are no monomers left. For this given set of conditions (temperature and concentration), therefore, the proteins in the aggregated state have just a probability of becoming monomers. If the number of proteins and the volume of the system are increased so that the concentration in unchanged (Fig 5 right) the probability of the proteins in the oligomers to go back in solution is the same as before but this time the probability of the aggregate to grow is also different from zero, because there is one more monomer left in the solution. As a result the stability of the oligomer of size *N* is increased. In other words, by increasing the number of proteins for a given concentration and temperature, the free energy barrier between the monomeric and the aggregated phases also increases and the stability of the aggregates increases as we can see for example from comparing the free energy at T = 0.4 in Figs 1 and 2.

Another reason for this change in stability is related to the change in the relative size of the fluctuations of the energy of the system. Being the energy an extensive quantity its expectation value (mean) is proportional to the number of particles in the system while its root mean square fluctuations (standard deviation) are proportional only to the square root of that number.
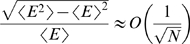
(1)


In small systems therefore, this ratio is not negligible, so the energy fluctuations can destabilise the system, while a system with large *N* having more degrees of freedom, has also more ways to absorb the energy and therefore its fluctuations are smaller. Small systems have larger spontaneous fluctuations and the stability of their aggregated phase changes consequently. This latter effect should be expected to be smaller for larger proteins [36] in which the large number of amino acids (degrees of freedom) will reduce, already in the monomer, the energy fluctuations. Indeed this would also be the case if the simulations are performed in explicit solvent as the number of degrees of freedom is increased and therefore the energy fluctuations become smaller.

### Finite Size Scaling

In Fig 5 we discuss the finite size analysis to this problem. This type of analysis is commonly used to estimate the typical systematic errors introduced by the finite size of the system on the calculation of various observables. The analysis starts by simulating systems of different sizes at equilibrium and calculating an observable (*e.g.* the internal energy). Following the procedure used in [38], we fit the average internal energy per peptide with an equation of the form: *e*(*N*) = *e*_∞_+*AN^α^*, where *e*_∞_ is the asymptotic value of the internal energy for infinite systems, N is the system size (*i.e.* the number of peptides or proteins used in the simulation) and α is the scaling exponent. For this particular example we do not have estimates for *e*_∞_ and α, as was the case in [38], therefore we fit all three parameters to the average internal energy calculated for the lowest temperature, T = 0.322, in which we know from our free energy profiles that the peptides are aggregated. Since we have three parameters to fit with only 6 points, our estimates will not be very reliable so by fitting our equation to all possible combinations of 5 points we obtain a rough estimate of their range of validity: −12.5≤*e*_∞_≤−9.5, 0.01≤*A*≤0.05, −1.1≤*α*≤−0.6. These values are obviously too broad and a more precise estimate, in particular of the exponent α, should be obtained, either by using a more coarse grained model that will allow simulations of large systems, or by an explicit analytical calculation. In Fig 5 we plot our data along with the best fit over the entire set of data as a function of *N* ^−0.82^, where −0.82 is the value of α taken from this fit. Future work will be required to understand more deeply the precise nature of the exponent α, particularly to understand if it belongs to the class of *critical exponents* [39] and has therefore a high degree of universality *i.e.* its value is constant for a class of different proteins having similar characteristics.

## Discussion

In the present paper we have discussed the how the number of protein present in the system, if small, influences the underlying free energy and therefore shifts the equilibrium between the different species. We have discussed two independent causes of this problem; the first being that the stability of an oligomer is related to the number of monomers or other oligomers still present in the system, the second related to the relative fluctuations of the system being proportional to *N* ^−1/2^.

The presence of this finite size effect has very important implications for the *in silico* studies of protein aggregation, *e.g.* it is the reason why usually the protein concentration in these studies is taken to be much larger than the concentration used in the corresponding *in vitro* studies and, more importantly, implies the presence of a systematic error in every estimate of the stability or the rate of formation of one specific aggregate configuration. A way to deal with these problems is to perform a finite size scaling analysis. This type of analysis allows to correct one observable (as we did for the internal energy in section “Finite Size Scaling”) for the systematic error introduced by the finite size of the system. We have outlined how this analysis works, how to carry it out, and we have estimated the values of the parameters within a small range. A more precise estimate of these parameters would be needed and to this end a coarse grained model should be employed to be able to study a much larger number of peptides and to obtain a precise estimate of the parameters of the scaling analysis. More work will also be required to investigate the degree of universality of such analysis *i.e.* whether a class of proteins with similar characteristics have the same critical exponent [39]. If verified this would represent a leap forward in our understanding of the generic physical properties of protein aggregation. The theory behind critical exponents was object of intensive studies in physics in between the 1964–1976. Nowadays, at least from a physical point of view the problem is essentially solved, although in many cases only approximate solutions are available.

Some biological problems require, however, the use of models with a detailed geometrical representation [15], [18], [20], [23], [40]. The use of these models makes the finite scaling analysis computationally very difficult, particularly for the study of large systems of medium or large peptides. In these cases a special care should be taken in the interpretation of the theoretical results. A useful approach is the one we have taken in a recent study [40] where we calculated the free energy as a function of the β-sheet size for two systems of 20 and 30 Aβ_25–35_ peptides, under the same conditions of temperature and concentration. A comparison between the free energy of the two systems allowed us to estimate up to which point our calculations were reliable, and the *trend* of the errors on the free energy due to the finite size effect. Understanding in detail the systematic error induced by the finite size effect in simulations of aggregation is becoming an issue of the utmost importance as theoretical studies are becoming more precise and their description of the process is becoming more quantitative. We believe therefore, that the results that we have presented in the present study and the results of [36] represent a very important initial step towards the formulation of a more general theory of finite size scaling for protein aggregation.

## Methods

Simulations were carried out with ProFASi (Protein Folding and Aggregation Simulator) [41], which implements an implicit water all-atom model [41]–[45] for protein folding and aggregation studies. The model assumes fixed bond lengths, bond angles, and peptide torsional angles, so that each amino acid has only has the Ramachandran torsional angles and the side chains torsional angles as its degrees of freedom. The interaction potential
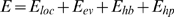
(1)is composed of four terms. The *E_loc_* term is local and represents an electrostatic interaction between adjacent peptides along the chain and the *E_ev_* term is an 1/*r* ^12^ repulsion between pairs of atoms. The hydrogen bonding contributions to the energy are calculated by a term, *E_hb_*, in which the distance dependence is modelled through a Lennard-Jones potential between pairs of *NH* and *C'O* groups within a given cutoff of 4.5 Å, and the angular dependence is expressed as a function of the 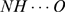 and 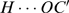 angles. The hydrogen bonds between the backbone *NH* (*C'O*) groups and the *C'O* (*NH*) groups on each side of them are disallowed. The GFF peptide used in this study, therefore, does not form intra-chain hydrogen bonds and the hydrogen bonding term of the energy monitors only the formation of intra-chain hydrogen bonds. The hydrophobicity term *E_hp_* is defined by a contact potential between hydrophobic side chains, the latter being proportional to the fraction of atoms in contact in the two amino acids. The parameters of the potential were chosen by optimizing the agreement with the melting temperature of the Trp. Cage mini-protein; the resulting force field has been shown to reproduce accurately the folded states and the melting temperatures of a range of polypeptide chains of both α and β structures, including Betanova, GB1p, LLM and F_s_, with excellent agreement with both CD and NMR data. In addition, properties such as the content of α-helix and the relative population of folded species was also found to be in excellent agreement with experimental data. ProFASi has also already been applied to study the aggregation of a series of short peptides, including the Aβ_16–22_ and Aβ_25–35_ peptides [15], [30], [40]. We performed simulations of six systems composed of three, four, six, eight, nine and twelve GFF three-peptides, using the parallel tempering technique [46]–[47] using 8 temperatures ranging from T = 0.322 to T = 0.588 (Fig 1, Fig 2 and Fig 3). We choose not to map the Monte Carlo temperature in Kelvin units as was done by Irbäck and co-workers [42] for the folding of small peptides because in the case of aggregation being the underlying free energy changed due to the finite size effect, it should be tested experimentally whether any such mapping still holds true. The simulations were performed in a cubic box with periodic boundary conditions of sizes respectively: 20, 22.01 25.2, 27.72, 28.84 and 31,74 Å. We changed the volume for different for systems with different number of peptides so to keep the concentration constant.
